# Issues in QT interval measurement

**Published:** 2004-10-01

**Authors:** Pallavi Lanjewar, Vaishali Pathak, Yash Lokhandwala

**Affiliations:** * Quintiles ECG Services, Mumbai; † Arrhythmia Associates, Mumbai

## Abstract

The QT interval, apart from clinical implications is crucial for safety assessment of new drugs under development. A QTc prolongation of even 10 msec in a study group is a warning signal for a new drug.

There are various issues involved in the measurement of the QT interval especially regarding the ending of the T wave and different morphological pattern of T-U complex. The other issue is significant spontaneous variability in the QT interval, resulting in spurious QT prolongation and unnecessary concern.

To minimize all these confounding factors, all clinical trials for assessing QT interval prolongation should be randomized and double blinded with appropriate control groups including placebo. ECG measurements should be done by  trained readers with electronic calipers at  ECG core Lab. ECGs should be compared  with multiple baseline values with multiple, time-matched on-treatment values.

 The QT interval represents the time required for completion of both ventricular depolarization and repolarisation and has been a parameter of particular interest [1]. Certain drugs have the ability to delay cardiac repolarization manifested as QT interval prolongation. This creates an electrophysiological environment that favors the development of cardiac arrhythmias, most important of which is torsades de pointes, but possibly other ventricular arrhythmias as well. This can result in sudden cardiac death [2]

Recently it has become evident that a variety of drugs induce QT interval prolongation. Besides the many antiarrhythmic drugs that are well known to widen the QT interval as their main pharmacological effects, non- antiarrhythmic drugs such as antipsychotic,  antiallergic, antibiotics and gastroenterologic agents have been shown to induce QT prolongation as a side effect. A careful assessment of QT interval prolongation is often required before novel drugs are approved by regulatory authorities. For accurate evaluation of the QT interval, the fact that the QT interval varies in RR interval dependent manner must be taken into account. Clinically, several QT-correcting formulas, such as Bazett’s, Fredericia’s, etc. have been used to evaluate QT prolongation considering changes in the length of the RR interval [3].

 The QT interval and corrected QTc are defined below: QT interval represents the duration of ventricular depolarization and subsequent repolarization, beginning at the initiation of the Q wave of the QRS complex and ending where the T wave returns to the isoelectric baseline.

## Choice of Lead

By convention Lead II is used for QT measurements. An alternative lead is used only when lead II was not adequate.

## Measurement of QT interval

 Often the end of the T wave is not clear. In such cases the end of the T-wave can be extrapolated by using the Tangent method. The start of the QT interval is defined as the first deflection of the QRS complex. The end of the QT interval is defined as the intersection of the descending part of the T-wave (positive T-wave) with the isoelectric line.

**Figure 1:**
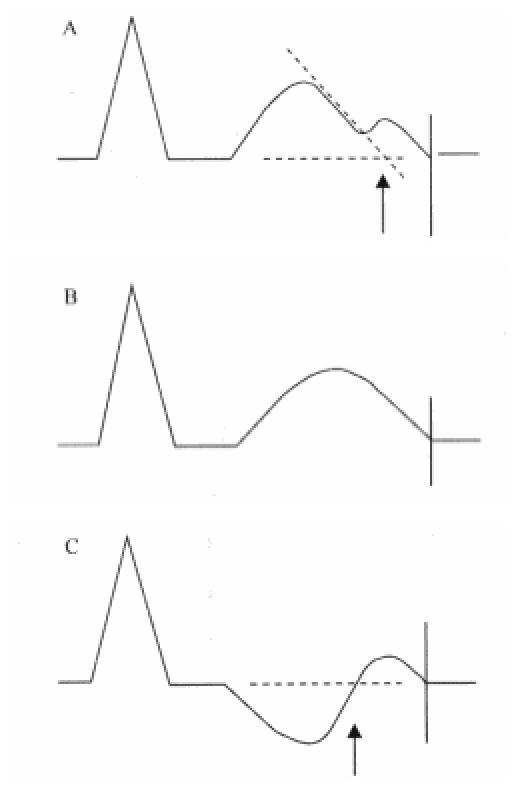
Schematic diagram showing QT interval. Three morphologic forms of TU complexes. Arrows indicate the end of QT interval measured excluding the second hump; vertical lines indicate the end of QT interval measured including the second hump.

 If a U-wave appears immediately after the T-wave has returned to the baseline, the QT interval is measured as the nadir between T and U-waves. If it is not clear whether a second hump occurs before the T wave has returned to baseline, it is included in the QT interval. (Figure 1)

QT interval for research purpose is measured by vernier caliper, digitizing pad on the paper ECG as well as by on-screen calipers in digital ECGs.

## Holter vs ECGs

A continuous 12-lead Holter can be used as an alternative method for the standard digital 12-lead ECGs because of following reasons i) It provides the  continuous ambulatory  recording for 24 hours, thereby enabling  retrieval of the ECGs based on pharmacokinetic data and (ii)It provides useful data for cardiac arrthymias by continuous beat to beat analysis [4]. However,12-lead Holter has many potential problems such as postural variation leading to change in ECG morphology [5-7] and multiple artifacts due to motion [8]. Due to the change in position of the limb electrodes in the Holter recording, there is significant change in the QT interval measurement as it is measured in Lead II and Lead II comprises of limb lead electrodes.

A study by Sarapa and colleagues [10] reviewed the utility of Holter-derived 12-lead ECG and standard digital 12-lead ECG in detection of sotalol-induced QT prolongation in healthy volunteers. They reported a difference between standard digital ECG and Holter-derived 12 lead ECG of 5.2 ± 19 ms, They also reported a difference of -5 ± 70 ms in measurement of the RR interval. The reason for this difference is apparently related to the    technique of recording or measurement at the ECG core laboratory.

##  Issues (fallacies of QT measurement)

### Wide QRS

The QT is less reliable when the QRS duration (QRSd) is ≥ 120 ms, as the increased QRSd which measures the duration of ventricular depolarization contributes to prolongation of the QT interval. The JT interval (defined as QT- QRSd) has been proposed as a more valid way to assess ventricular repolarization in such circumstances.

Zhou SH et al [11] examined the influence of QRS duration on the JT and QT intervals in 20,687 normal adult subjects and 2,865 subjects with various categories of intraventricular conduction delay (IVCD). Estimates for coefficients for multiple regression of QRS duration on QT and JT intervals combined with a correction term for heart rate (HR) were determined for each VCD category. QRS duration accounted for about 16% of total QT variation, but had a practically negligible effect on JT interval in complete bundle branch blocks. A single-parameter formula was derived for the JT prolongation index of the form JTI = JT (HR + 100)/518, with a JTI > or = 112 identifying repolarization prolongation in all VCD categories. They concluded that it is preferable to use JT rather than QT as a more appropriate index of duration of repolarization in IVCD.

Das G [12] evaluated the relative contribution of the depolarization and the repolarization time prolongation to the prolonged QT interval in patients with (IVCD). The QRS, QT, and JT intervals were measured in 72 subjects with various types of IVCD. The observed intervals in IVCD subjects were compared to similar intervals in 33 healthy individuals in whom there was no evidence for intraventricular conduction abnormalities. The QTc (QT interval corrected for heart rate) in subjects with IVCD were 445 ± 6.8 msec (mean ± SEM) in those with LAD, 470 ± 9.1 msec with RBBB, and 489 ± 6.9 msec with LBBB. All of these intervals were significantly prolonged compared to 430 ± 4.3 msec in the control group. The prolongation of QTc interval in each category of IVCD subjects was entirely secondary to a prolonged depolarization time, as the JT intervals were not significantly different from those observed in the control group (F = 0.5, p = NS). These observations may provide an explanation for the differential prognosis for subjects with prolonged QT interval with prolonged repolarization time as compared to those with prolonged QT interval with prolonged depolarization time.

Prolonged heart rate-corrected QT interval is associated with higher risk of mortality in patients with coronary heart disease (CHD). Crow RS et al [13] assessed JT interval in these patients with CHD and QRSd  ≥ 120 ms. and concluded that the JTc is a simple measurement that is a significant independent predictor of  CHD events in men with wide QRS complex.

### Gender: 

Bazett’s formula (QTcB) has been more frequently used in the medical literature than Fridericia’s formula, so that most reported criteria for normal and abnormal values are derived from Bazett’s formula (Table 1) [14]

### Magnitude of QT variability

Significant spontaneous variability is seen in the QT interval, and this may result in spurious QT prolongation and unnecessary concern about cardiac safety. To avoid this spurious variability, data on QT, QTc intervals should always be presented both as analyses of central tendency  (means, medians, ranges, etc.) and categorical analyses (proportion of individual subjects in each  treatment group experiencing specified degrees of abnormality i.e. outlier analyses).

## Analysis of central tendency

For analyses of central tendency, the emphasis should be on following factors

###  Maximum change in the QT, QTc Intervals

The maximum observed difference  between on-treatment and baseline QT, QTc values should be expressed both as mean  and median changes in the population. This value is meaningful only as a comparison  with placebo or a non-QT prolonging drug, as selection of the highest of many on- treatment values will invariably show an increase from baseline.

### Time matched QT, QTc Intervals

Mean changes from baseline in the observed QT, QTc interval can be presented as time-matched control and treatment group values. Time matched ECGs counter the circadian variation in the QT interval. Although these values may show regression to the mean, they do not have the same upward bias as the maximum change.

## Categorical analyses

As the absence of statistically or clinically significant differences between the test drug and  comparator groups does not exclude the possibility of marked QT,QTc interval prolongation  occurring in individual subjects, analyses of central tendency should always be accompanied by  appropriate categorical analyses.

Categorical analyses of QT, QTc interval data are based on the number and percentage of patients meeting or exceeding some predefined upper limit value. Clinically noteworthy signals may be defined in terms of absolute (readings in excess of some specified threshold value) QT, QTc intervals or changes from baseline control Although increases from baseline in the QT, QTc interval constitute signals of interest, interpretation of these differences is complicated by the potential for changes not related to drug therapy, including regression toward the mean and choice of extreme values. Regression toward the mean refers to the tendency of subjects with high baseline values to have lower values at later time points, while subjects with low baseline values tend to experience increases. The direction of regression depends on initial selection criteria; e.g., if subjects with high baseline QT, QTc interval values are excluded from the trial, values recorded during treatment will tend to rise relative to baseline levels.

The process of choosing the highest of multiple observed values will also invariably cause an apparent change from any single baseline value, a phenomenon found in both drug and placebo-treated groups.

To minimize the effect of spontaneous variability, we put forward the following recommendations: 1) To use trained readers2) To take on-screen measurements in an ECG core Lab 3) To compare with the results in the appropriate control group(s), including placebo or a drug with no QT, QTc prolongation effect 4) To compare with multiple baseline values with multiple, time-matched on-treatment values (not just the greatest value).

## Figures and Tables

**Table 1:** 

Rating	QTcB in msec	
	Adult Males	Adult Females
Normal	< 430	< 450
Borderline	430-450	450-470
Prolonged	> 430	> 450

## References

[R1] Kautzener J (2002). QT interval measurements. Card Electrophysiolol Rev.

[R2] Lazzara R (1993). Antiarrhythmic drugs and torsade de pointes. Eur Heart J.

[R3] Hassimoto Masatoshi, DVM (2002). Accurate Evaluation of QT Interval in conscious Rhesus Monkeys by use of Holter ECG. J of Electro cardiology.

[R4] Denes P (1992). The importance of derived 12-lead Electrocardiography in the Interpretation of Arrhythmias detected by Holter recording. Am Heart J.

[R5] Kowey KR, Kocovic DZ (2003). Ambulatory Electrocardiographic Recording. Circulation.

[R6] Ng J, Sahakian AV, Swiryn S (2003). Accelerometer-based Body- position sensing for     Ambulatory Electrocardiographic Monitoring. Biomed Instrum Technol.

[R7] Shiner Z, Baharav A, Akselrod S (2003). Detection of different Recumbent Body positions from the Electrocardiogram. Med Biol Eng Comput.

[R8] Jones AY, Kam C, Lai KW (2003). Changes in Heart rate and R-wave Amplitude with Posture. Chin J Physiol.

[R9] Bethge KP, Gonska BD (1985). Long-term electrocardiography: value and reliability of various systems. Z Kardiol.

[R10] Sarapa N, Morganroth J, Couderc J (2004). Electrocardiographic identification of drug-induced QT prolongation: assessment by different recording and measurement methods. Ann Noninvasive Electrocardiol.

[R11] Zhou SH, Wong S, Rautaharju PM (1992). Should the JT rather than the QT interval be used to Detect Prolongation of Ventricular Repolarization? An Assessment in Normal Conduction and in Ventricular Conduction Defects. J Electrocardiol.

[R12] Das G (1990). QT interval and Repolarization Time in Patients with Intraventricular Conduction Delay. J Electrocardiol.

[R13] Crow RS, Hannan PJ, Folsom AR (2003). Prognostic Significance of Corrected QT and corrected JT interval for incident Coronary Heart Disease in a general population sample stratified by presence or absence of wide QRS complex: the ARIC Study with 13 years of follow-up. Circulation.

[R14] Moss AJ (1999). The QT interval and torsade de pointes. Drug Safety.

